# Kombucha: Perceptions and Future Prospects

**DOI:** 10.3390/foods11131977

**Published:** 2022-07-04

**Authors:** Patrícia Batista, Maria Rodrigues Penas, Manuela Pintado, Patrícia Oliveira-Silva

**Affiliations:** 1 Human Neurobehavioural Laboratory, Research Centre for Human Development, Universidade Católica Portuguesa, Rua de Diogo Botelho, 1327, 4169-005 Porto, Portugal; mariarodriguespenas@gmail.com (M.R.P.); posilva@ucp.pt (P.O.-S.); 2 CBQF—Centro de Biotecnologia e Química Fina—Laboratório Associado, Universidade Católica Portuguesa, Escola Superior de Biotecnologia, Rua de Diogo Botelho, 1327, 4169-005 Porto, Portugal; mpintado@ucp.pt

**Keywords:** Kombucha, nutraceuticals, legislation, gut-brain, health

## Abstract

Background: Kombucha is an increasingly consumed product classified as a nutraceutical. Legislative efforts about these products remain confusing and without global harmonization. This natural product has been developed to improve or promote physical and mental health. However, it needs regulatory guidelines to control the production and guarantee the product’s efficacy and safety. Aim: The study intends to draw attention to the need for regulatory guidelines and the potential of this product in the market and peoples’ health. Key findings and conclusions: The lack of regulation and the low level of literacy about this product can limit its development, marketing, and impact on health. Thus, it is essential to highlight the potential value of this product and invest in its development and marketing. Likewise, it is important to spread awareness among the population of these products and their impacts on people’s health. Thus, this study focuses on a pertinent theme and alerts to the need for legislation for these products, to draw attention to the inexistent legislative control and the consequent need for regulatory guidelines for better and safer production and consumption.

## 1. Introduction

In the light of society’s evolution and modernization, there is the aspiration and idealization of a healthier community. For this, nowadays, there is a growing awareness of sustainable policies to promote the quality of life of the population and active and healthy aging [1].

Food is one of the pillars of health. Reconfiguring food systems, and introducing new policies in line with the Sustainable Development Goals (SDG), is extremely important to deliver healthy and sustainable diets and new products to everyone in order improve their lives and the planet [2,3,4]. Another significant aspect of this reconfiguration process is to adopt a healthier diet based on more beneficial products, which is fundamental to reduce mortality and morbidity and improve quality of life. On the other hand, an unhealthy diet is responsible for micronutrient deficiencies or dietary excesses, and it contributes to creating and increasing the incidence of diet-related diseases or conditions, such as obesity, cardiovascular diseases, diabetes, cancer, and other pathologies [5].

It is expected that circular food-related technology, based on the 4R strategies of reducing, reusing, recycling, and recovering, will positively affect several SDGs [6]. There is a growing interest in food technologies to develop functional and bioactive ingredients for health promotion and guarantee food safety [2,4]. For example, the study of microbial protein has increased prominently and includes algae, yeast, bacteria, and fungi. Furthermore, functional food research, i.e., a rising study field aiming to explore natural active components that provide health benefits beyond essential nutrition, has advanced drastically to prevent diseases and promote wellbeing and people’s quality of life. Functional foods are products that have the necessary nutrients for human survival, providing some health benefits related to physical and mental health [7,8]. Some functional foods examples include probiotics, prebiotics, dietary supplements, vitamins, and antioxidants [7].

Nowadays, particular emphasis is being placed on including probiotics or prebiotics in the diet [7]. Probiotics are defined as a group of microorganisms (such as *Bifidobacterium*, *Lactobacillus*, *Zygosaccharomyces*, and others) that, when consumed in adequate amounts, provide some health benefits to the host organism, like boosting the immune system [9] and balancing the intestinal microbiota [10]. They have also been reported in the literature as a way to prevent or even treat some diseases, such as cancer [11], cardiovascular diseases, diabetes, and allergic reactions [12].

Probiotics have been integrated into products or food supplements, highlighting the incorporation in fermented dairy products [7]. However, more recently, they have been incorporated into a non-dairy product that has gained popularity, known as Kombucha. This product is being developed and commercialized in different countries. Still, more investigation is needed to guarantee the quality of the production process and safety, because there are no regulatory guidelines available in Europe [8].

Therefore, this study intends to draw attention to the need for regulatory guidelines and the potential of this product in the market and people’s health.

## 2. Kombucha: Concept and Health Benefits

Kombucha originated in China, through traditional processing. Then, it was disseminated to Japan and spread through Russia and eastern Europe, gaining worldwide popularity during World War II [13,14]. Then, it broke into western societies and, more recently, into Europe and the United States of America (USA).

This product is a non- or low-alcoholic functional beverage, slightly acidic and carbonated. It is obtained by the fermentation of sweetened tea with probiotic microorganisms, called SCOBY (symbiotic culture of bacteria and yeast). The manufacturing process consists of SCOBY fermentation during 7–21 days [13]. A higher fermentation time helps increase the amounts of bioactive compounds, like polyphenols; B vitamins; vitamin C; and essential minerals like Fe, Mn, Zn, Cu, and Ni [15]. In fact, phenol and flavonoid compounds decrease at the beginning of fermentation and increase throughout it [16]. Even though the higher fermentation time is associated with the formation of these compounds, which are highly beneficial for human health, the increased fermentation time also augments the production of organic acid by SCOBY. Thus, it is necessary to follow the recommendation time reported in the literature to ensure the safety of the consumer [17].

**Figure 1:**
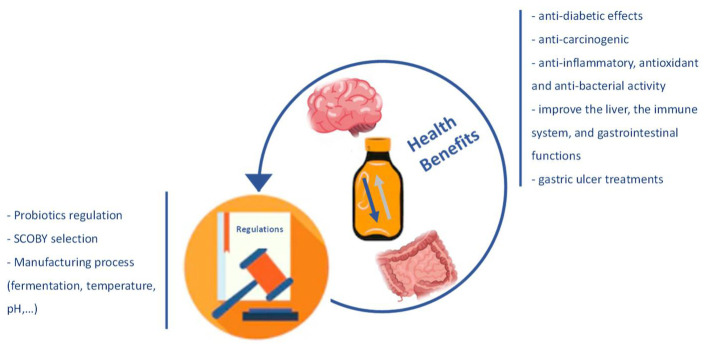
Description of the Kombucha’s health benefits and parameters lacking clear regulation worldwide.

The microorganisms most associated with the probiotic function are bacteria such as *Lactobacillus*, *Bifidobacterium*, *Bacillus cereus*, *Propionibacterium freudenreichii*, and yeasts of the genus *Saccharomyces* [18,19]. These microorganisms present benefits in terms of the health microbiome, but they also produce other relevant compounds, such as amino acids, organic acids, sugars, polyphenols, and vitamins. Some other micronutrients, also resulting from the fermentation process, foster Kombucha’s potential benefit [13,20]. Several in vivo or in vitro studies, especially with animals and, more recently, with humans, have reported some benefits of this product. There are many health-promoting effects reported in the literature, such as anti-inflammatory, antioxidant, anti-bacterial, anti-diabetic, anti-carcinogenic activity; reducing the concentration of cholesterol; and improving the liver metabolism, the immune system, and gastrointestinal functions [21,22,23,24] (Figure 1).

The literature also highlights the high antioxidant potential of Kombucha [21,24,25]. This antioxidant property depends on three important factors: the type and composition of the tea infusion, the SCOBY, and the fermentation process. For example, green tea present in most Kombucha compositions already stands out with, significant antioxidant properties, but the fermentation process may promote an increase in the antioxidant potential of Kombucha [24]. Polyphenols present in kombucha are responsible for antioxidant activity. In fact, during the Kombucha fermentation process, there is an increase in specific phenolic compounds, and the fermentation time has an influence on the rise and the content of these compounds. Phenolic compounds are also involved in anti-inflammatory properties. In vitro studies have shown an improved inhibition of some enzymes responsible for inflammation by Kombucha relative to unfermented tea [22]. The phenolic and flavonoid compounds have hydroxyl groups that help to stabilize free radicals, inhibiting their formation and decreasing the degradation of collagen and hyaluronic acid, which can deaccelerate the skin aging processes [16,26]. Furthermore, the low pH and bacterial cellulose found in SCOBY inhibits bacterial action on the skin and stimulates cell regeneration [27]. It is important to clarify that Kombucha’s SCOBY has not been used exclusively for the preparation of Kombucha, but it has also been used for the adsorption of heavy metals or for the textile industry, for example [13].

On the other hand, the anti-carcinogenic activity seems to depend on the antioxidant and anti-inflammatory activities, mainly associated with an antiproliferative effect [25,28]. For instance, Villareal-Soto and colleagues [23] tested Kombucha tea extracts against human breast cancer and human colon cancer. Their results showed increased inhibition of cancer cells’ proliferation after the continued consumption of Kombucha.

In addition to phenolic compounds, other important compounds resulting from the fermentation process are organic acids (e.g., acetic acid, gluconic acid, and glucuronic acid). The antimicrobial activity is primarily associated with the organic acids present in Kombucha, particularly acetic acid [25,29]. Studies showed that kombucha is effective as an anti-bacterial agent against *Staphylococcus aureus*, *Staphylococcus epidermis*, *Pseudomonas aeruginosa*, *Klebsiella pneumoniae*, *Escherichia coli*, *Proteus vulgaris*, *Proteus mirabilis,* and *Bacillus subtilis* [29,30,31].

Kombucha has also been significantly associated with gastrointestinal and liver effects [13]. One way to explain these effects is because the probiotics present in Kombucha may affect the composition and function of the intestinal microbiome [32]. On the other hand, the hepaprotective efficacy of kombucha has been studied by liver toxicity markers’ assessment, such as serum glutamic pyruvate transaminase, assessing the antioxidant enzymes, like superoxide dismutase and catalase, and various levels of creatinine and urea in the liver tissue [25]. Lee and collaborators [33] were able to show the protective characteristic of Kombucha in insulin-resistant mice, improving liver function, restraining hepatocytes apoptosis, and protecting their metabolism, reducing liver inflammation and fibrosis.

Some earlier studies have also reported the potential benefits of Kombucha on longevity. Additionally, more recently, modern studies have brought new interest in the impact of probiotics on the gut–brain axis [34,35] and on this bidirectional communication, suggesting that more rigorous future trials are needed to draw firm conclusions about the benefits associated with fermented food in healthy aging and longevity [36,37].

## 3. Kombucha Market and Safety

The interest in natural products and nutraceuticals has increased because people are more interested in their bodies, health, and aging [7,38,39,40]. In the same way, the Kombucha market is growing and becoming quite famous globally, including family businesses (small businesses), which have gained considerable prominence in this field. It is estimated that there are approximately 235 companies of Kombucha distributed through Europe, North America, and Asia [8]. The market should reach USD 3.5–5 billion by 2025 [8]. This market increase shows that more and more people are consuming Kombucha, but there are still some unsolved questions that may leave the consumers a bit suspicious if not elucidated. The lack of information about the product, its quality, and safety may condition the purchase and consumption. Therefore, increased literacy in this area is necessary, as well as the definition of regulations that guarantee the quality standards, efficacy, and safety of the product.

Since Kombucha is a beverage that contains probiotics, some care is needed during the production and conservation process to guarantee the product’s quality and expected benefits. The literature reports cases associated with health problems resulting from the consumption of this beverage, such as dizziness, headache, allergic reactions, and gastrointestinal toxicity [13,20,41]. These problems are due to several factors, such as overconsumption; poor hygiene conditions during the preparation (more associated with homemade Kombucha); incorrect fermentation (e.g., long fermentation leads to a very acidic drink); incorporation of unbeneficial bacteria or inaccurate selection of SCOBY; and consumption by patients that are immunocompromised, such as people with health problems or that are pregnant [13,42]. For example, pregnant women also have been advised not to consume Kombucha, not only because of the possible presence of alcohol, but also due to the possible presence of heparin, as it inhibits the blood clotting system’s proteins and can increase the chance of hemorrhages [20]. Another example is that excessive consumption by adults can be harmful. According to the Centers for Disease Control and Prevention (CDC), the daily consumption of 100 grams of Kombucha does not pose a health risk to the drinker, but excessive consumption (<340 grams daily) can be risky, and cases of metabolic acidosis have been reported [20]. Therefore, it is important to know the recommended limit to avoid adverse reactions.

Some of the reported health problems are due not only to excessive consumption, but also to the processing methodology used to produce Kombucha. Home production is often reported due to the lack of hygienic conditions in the production of the products, in which certain pathogenic microorganisms can contaminate the product, as well as the use of improper containers for its production (e.g., glass bottles are preferred) [8,41]. Furthermore, there have been reported cases of gastrointestinal toxicity and lead poisoning related to contaminants that came from the enamel pigment on the vase containing the Kombucha [20]. The production process is critical because it involves several factors that may compromise the quality of the final product. Despite the microbiological composition, diversity, and quantity, other parameters such as the substrate, the temperature, the pH, and the production container are also important [43]. Thus, safety requirements are needed to control the production and assure the consumer that the product is, in fact, safe for human consumption.

## 4. Consumer’s Acceptance of Kombucha

In fact, there is an increase in functional and fermented foods consumption, because consumers tend to support new trends to improve their health [8]. As a result, businesses in this area have grown to almost 2 billion euros in 2019 (Kombucha Brewers International, 2019). Regarding Kombucha, authors like Walker (2021) found that the main reason why people are interested in Kombucha is connected to the supposed benefits of regular consumption: boosting the immune system, preventing cardiovascular diseases, and antidiabetic and anticarcinogenic activities, among others [23,44]. In consequence, consumers are demanding more scientific evidence about the potential benefits. Even though Kombucha is considered a natural and healthy product, many nutrition claims still lack scientific proof [45]. There is clearly a significant growth in the Kombucha market [8], especially in the older generations [44]. It is important to increase health literacy on awareness of healthy dietary habits, especially among young consumers [44,46].

Although some countries have published guidelines to ensure Kombucha quality and safe production, the lack of legislation and the use of unfounded health claims constitute obstacles to further Kombucha’s popularity among consumers. Consumers usually like to follow health trends, especially when natural products and health benefits are involved [47]. However, in order to maintain or increase consumers’ interest and acceptability of Kombucha, at this point, it is necessary to provide medical advice that supports the credibility of those benefits [48]. Because of that, governments are being pressured to put in place specific legislation to ensure product safety. This way, people will trust and be willing to buy products with human health benefits claims.

## 5. Legislation and Regulation of Probiotics

There has been an increased production of functional foods, involving costs and risks [49]. The development of a new product and its marketing must follow strict criteria that control the processing and guarantee the quality of the product [43]. Within the framework of functional food, beverages occupy the most active position, with a particular highlight for sport drinks, energy drinks, and probiotic drinks, such as kombucha [8].

The commercialization of an industrial product must be well regulated to avoid problems that compromise consumer safety. Today, Kombucha is no longer an artisanal product, which was in the past conducive to the development of unsafe practices that could compromise the quality of the product, but it is a product that is increasingly expanding in the industry [8]. Regarding the number of industrial companies in the world producing Kombucha, Europe has 30 companies registered, Asia Pacific Region has 31, North America has 162, and Latin America has 12 companies [8].

**Figure 2:**
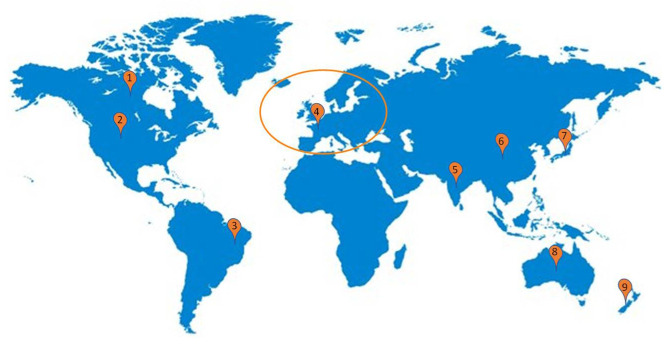
General probiotics regulatory guidelines reference worldwide: America (1, 2, 3); Europe (4); Asia (5, 6, 7); Oceania (8, 9).

Kombucha regulation, or nutraceuticals regulation in general, is very scarce (Figure 2). Similarly, western societies do not have proper legislation regarding functional foods or nutraceuticals, because these products are seen as a concept rather than a food category [50]. Thus, due to the lack of regulation, and to help producers and consumers understand what parameters need to be carefully assessed in Kombucha manufacturing, a non-profit organization called “Kombucha Brewers International” was created [8,43].

Parameters such as the inoculum, the recipient, the refrigerated distribution and storage, flavors, unpasteurization, and the fermentative process are parameters that need clear and standard criteria for its commercialization [8,43].

Kombucha and other probiotic products need a more regulated guidelines to prevent contamination or health risks due to the manufacturing process and ensure the quality of these products [51]. Unfortunately, the regulations regarding probiotics are imprecise, which puts at risk the consumer and the producer’s manufacturing processes [51]. This lack of regulation can also be associated with a wide range of concepts to describe probiotics. There is not a consensus definition of what probiotics are or which background they come from, which generates confusion in the legal requirements for the acceptance of these products. This means that every country or continent has a different definition for probiotics, known as a functional food in Japan, China, and Malaysia; as a food supplement in Sweden, Denmark, and Finland; and biotherapeutic/pharmaceuticals in Belgium and Germany [52].

### 5.1. Asia

#### 5.1.1. Japan

Japan is the market leader in probiotics [7]. The use of fermented products is part of its traditional gastronomic culture, and citizens are encouraged to adopt healthier lifestyles. Furthermore, Japanese hospitals have been using probiotics for some gastrointestinal disorders treatments, such as diarrhea and constipation, and other health conditions, like high blood pressure and diabetes [53].

In 1991, a regulatory system was created, “Foods for Specific Health Uses” (FOSHU), for functional foods, in which probiotics were included [7,53]. Food claims are divided into categories depending on the scientific evidence [52].

In Japan, the regulatory classification system distinguishes probiotics according to their use: drugs or non-drugs [7,53]. Probiotics can be used in medicine, but most of them are food products, like yogurts, or foods with health claims (FHC), like kombucha. FHC can be categorized as “foods with nutrient function claims” (FNFC), “foods with specific health use” (FOSHU), or “foods with functional claims” (FFC) [7]. There is no specific restriction associated with probiotic food; however, fermented milk or lactic acid drinks are required to have a lower limit of LAB (lactic acid bacteria) [53]. In addition, producers need special permission from the Ministry of Health and Welfare (MHLW) for the product to be considered a FOSHU, and they are not allowed to show efficacy claims labels [51,53]. FOSHU registration depends on the safety, efficacy, processing, and analytical parameters as well as other important information associated [52].

#### 5.1.2. China

China is one of the world’s most developed markets for nutraceuticals, and just like Japan, this popularity is mainly due to traditional gastronomic culture [54]. In 1995, the Food Hygiene Law was created to approve health foods licensing [55]. The Chinese government has categorized nutraceuticals into ‘dietary supplements’, ‘new resource food products’, and ‘functional foods’ in order to better organize these new products [55].

The ‘dietary supplements’ are the common vitamins, minerals, amino acids, and probiotics, among others, whose role is to provide the essential nutrients, prevent nutrient deficiency, and reduce the risk of developing certain diseases. According to the Food Safety Law, these supplements must have the “blue hat” label and be truthful to their claim [55].

The ‘new resource food’ category was created to motivate the search for new materials to be added to the food list. Unlike functional foods, these products do not need scientific proof of their efficacy if there is supportive literature indicating benefits and safety to human consumption [55]. Its approval process is similar to functional foods. As for functional foods, health foods [50], or foods with specific health functions, the detailed description of each product must be submitted to the department of public health for approval (article 22, Food Hygiene Laws) and, likewise, must be safe to human health (article 23, Food Hygiene Laws) [55]. The China Food and Drug Administration (CFDA), the former State Food and Drug Administration (SFDA), is responsible for conducting all affairs regarding functional foods. Since this function encompasses any type of supplement with differential health functions [50,54,55], the CFDA has a vital role in the control of raw materials and ingredients included in food products. Health claims are associated with functional foods, and they are classified into two types: ‘nutrient claims’, or a supplier of a particular nutrient, and ‘general functional claims’, which refer to assisting in health improvements [50]. Currently, there are 28 health functions that can be claimed [7]. Regarding its selling, the label should include all the non-misleading information regarding the product: analytical; chemical and physical parameters; total and solid weight; health function; and a blue hat symbol, which serves to distinguish the product from drugs [55].

Probiotics fall under this last category, and some strains have been specifically approved to be used in functional foods, such as *Bifidobacterium animalis* and *Streptococcus thermophilus* [7,54]. However, even though China has a specific list of microorganisms that have been specifically approved for usage in functional foods, there are no specific requirements regarding probiotics [54]. As for claims, there is no limit on which health claims can be used in these products, especially for functions like “enhance immunity” and “regulate gastrointestinal flora”. However, products that contain probiotics and aim to have health claims require an 18–24-months-long pre-market registration [7]. It should be noted that Kombucha falls under the category of probiotics, having no specific regulation that controls its production and selling.

#### 5.1.3. India

In India, probiotics are regulated according to their function. In other words, it depends on if they are characterized as food or drugs, usually falling under the food category [56]. The Indian Council of Medical Research (ICMR) was created due to the lack of regulating guidelines for the sale of probiotic products. This entity controls and assesses the efficacy, safety, and health claims associated and labelling issues [52,56,57]. The ICMR provides guidelines and evaluates the species and strains that constitute the product; assesses the potential probiotic activity and possible toxicity of the strain; evaluates the efficacy in animal models, the safety for human consumption, and the minimum dosage to have the desired health benefits; and finally, specifies the label requirements regarding presenting all the information regarding the product [57].

Most frequently, probiotics are categorized as functional foods [56] and are regulated by the Prevention of Food Alteration (PFA), Food Drug Administration (FDA), and Food Safety Standards Act (FSSA). Those institutions define foods for their functionality and enact the ICMR guidelines [7,56,57]. For instance, the FSSA was created with the mission of ensuring the availability of specific regulations for nutraceuticals, foods, and dietary supplements [56,57]. However, even though there is a legal definition for functional foods, a specific categorization and the minimum standard safety are lacking [57]. The PFA oversees the labelling information and packaging, requiring the maximum information about what is being sold [56]. Nevertheless, it is still needed to clarify the regulatory framework, especially at the manufacturer’s level. Due to the increased consumption of probiotics, companies may be using strains, and some of those strains are even novel in the market, and the safety of production and consumption needs to be assured [56].

### 5.2. Europe

Despite Asia and the Pacific region dominating the functional food and probiotic market, Europe presents a promising market, where the regulations regarding health claims have shown a slow growth due to the health claim regulations in the Europe Union [7].

The consumption of probiotics in Europe occurs mainly in foods and food supplements, especially dairy foods and dietary supplements [58]. Furthermore, Europe was the second to have a definition for functional foods and beverages. However, this regulation seems very confused [7,58].

In Europe, probiotics are covered by the Food Product Directive and Regulation (regulation 178/2002/EC; directive 2000/13/EU). In the case of the probiotic products, they have been commercialized as herbal products, and the Herbal Medicine Products Directive (2004/24/EC) has required that all microbial products apply and obtain a drug registration, or they will be marked in the food category, where it will have to obey to the regulation of food supplements [58]. This regulation is supported by the European Food Safety Authority (EFSA), which evaluates the safety of any food or food ingredient, and by the entity Functional Food Science in Europe (FUFOSE), which regulates the functional foods category, including the European probiotic market [7,59]. However, even though it was the second region to have a definition for these and other functional foods and beverages, at the present moment, Europe forbids the use of the term “probiotics”, demanding that all health benefits associated with the consumption of probiotics be scientifically proven and authorized before being claimed, implemented by the European Union Commission and EFSA [7,51].

The evaluation of nutritional and health claims is under Regulation number 1924/2006, and it is required that the product is manufactured according to scientific evidence, keeping products with unfound claims off market, keeping food consumption safer [59,60]. In addition, all probiotic products, since they contain microbial culture, must go under a Qualified Presumption of Safety (QPS) assessment test, which is a commission responsible for providing a more concrete generic safety assessment on microorganisms that are intentionally used in foods and food supplements, acting according to EFSA’s scientific panels [61,62]. The EFSA has issued a list of microbial cultures with a QPS symbol, meaning that it no longer requires any safety test assessment [51]. A typical concern in this field is that the degree of control of the manufacturing process is insufficient. One example is that since the term “probiotic” is not allowed on the label, consumers are faced with the microorganism’s Latin term on the product, which creates some confusion about the purpose of the product, because it demands a certain knowledge by the consumer about the product [7,51]. Nonetheless, since probiotics have been traditionally used in Europe, consumers do not fear the consumption of bacteria if it is used in the traditional forms of dairy products. However, it is an obstacle for new foods, such as Kombucha and kefir, because it makes the evaluation of these products and their health properties a bit more difficult.

### 5.3. America

#### 5.3.1. Brazil

There has been a growing market for probiotic products in Brazil and an increase in the citizens’ awareness of their health benefits [63]. Brazil was the first American country with functional food legislation, where the Minister of Health assesses the health properties and the specific strains used as probiotics through the Agência Nacional de Vigilância Sanitária (ANVISA) [52,63]. In the late 90s, ANVISA established the standard criteria to regulate functional foods in Brazil [64]. Later, ANVISA started to require an informed label with the ingredients, functional claim, and product lot number information [64]. According to ANVISA, the probiotic products were categorized into infant and enteral foods, simple supplements, and supplements with added vitamins and minerals [63].

In 2018, ANVISA started to require proof of the safety and health benefits of the strains used [63]. Furthermore, it made available a complex regulatory system for functional foods. The new legislation of 2018 requested that the companies identify the probiotic strain, by genotypic and phenotypic information, to support the physical or mental health-related benefits claim [63]. General claims only require a clinical study with proven evidence of the beneficial effect, but specific claims are for new claims and need further studies to verify their safeness.

Concerning Kombucha, this product has gained massive popularity in Brazil, being the first country with Kombucha legislation (Instrução Normativa Nº41) [65], where this probiotic product is defined and classified. The Kombucha regulation specifies what information the label must contain, the mandatory and optional ingredients, the maximum value of alcohol that may be included, and what other analytical parameters must be assessed during the production. For example, the denomination of the beverage must be as follows (Table 1): Kombucha (ingredients and strains used, if any, before the fermentation with *Camellia sinesis*) with (optional authorized ingredients) with flavor (name of the additive) sparkling (if with sparkling water) with alcohol (if any above the 0.5% *v/v*) [65].

#### 5.3.2. United States of America

The probiotic market in the USA is not very wide, and most probiotic products are imported from other countries [66]. In the USA, probiotics are categorized mainly as food or as dietary supplements. In order to monitor these products, two commissions were elected: the Dietary Supplement Health and Education Act (DSHEA) and the FDA, to regulate probiotics by their functional activity and characteristics [51,67]. If the probiotic is considered a dietary supplement, it will be under DSHEA and FDA control, but if it is perceived as food, it will be regulated by the FDA [67]. The United States FDA is responsible for the safety, labelling, and health claims of food and other supplements [66]. Under the regulation of the Food Drug and Cosmetic Act (FDCA) any substance intentionally added to food is subjected to review and approval by the FDA, excluding the ones that are recognizable to be safe under the conditions of its intended use [59].

Similar to the European QPS, probiotics must be regulated under the same regimen as other food ingredients and be given the Generally-Recognized-As-Safe (GRAS) status in the USA, which depends on the scientific evidence that shows the safeness of the microbial [59]. A probiotic integrated in a food or dietary supplement does not need an FDA notification, as long as it was not chemically altered or claimed to prevent, diagnose, or treat any disease [59,67]. Unlike Europe, producers can make structural, nutritional, and health claims in the USA, as long as they are accompanied by an FDA-mandated disclaimer showing truthful and substantiated information supported by scientific evidence [7,51].

In the specific case of Kombucha, when this product suffers alcoholic fermentation, the federal law indicates that non-alcoholic beverages should not contain more than 0.5% alcohol by volume at any time during production, bottling, and selling [8]. If the alcohol by volume level is superior to 0.5% at any moment, Kombucha will be regulated by the Alcohol and Tobacco Tax and Trade Bureau [8]. In the meantime, Kombucha manufacturers are trying to pass a bill that exonerates the federal alcohol taxes, as long as alcohol Kombucha usually does not exceed t1.25% alcohol (Keeping Our Manufacturers from Being Unfairly taxed while Championing Health Act—Kombucha Act [68]).

#### 5.3.3. Canada

Until now, Canada has not had a solid structural regulatory framework for probiotics [52]. As a result, probiotic products are mostly considered natural or homeopathic products, presented as products capable of providing pharmacological or other health benefits [52]. Probiotics and their packaging regulations fall under the Natural Health Products Regulations. These regulations are set by the Canadian food and Drug act [52]. The Canadian Food Inspection Agency (CFIA), alongside the Food and Drugs Act and Food and Drug Regulations, assesses the safety and stability of probiotic food products through guidelines specified by the “Guidance Document—The Use of Probiotic Microorganisms in Food”. These entities also control the labelling information and the ingredients list used in the production process and what type of strains are safe for human consumption [69].

There are three types of claims made about probiotic products: health, function, and therapeutic claims [69]. Health claims refer to the suggestions that a food component may affect people’s health. Compared to the health claims, a function claim presents a more detailed explanation of the health benefit mechanism [69]. On the other hand, the therapeutic claim presents probiotics as natural health products. In Canada, probiotics are most frequently shown with the therapeutic claim and are regulated by the Natural Health Product Regulations [70]. Both health and function claims must obey the regulatory guidelines of CFIA [70]. For both claims, it is recommended that the term “probiotic” be accompanied by a specific and validated statement on the beneficial effects and a specific report on the physiological effects of the probiotics [69]. This regulation seems to have some gaps due to the conception of probiotic products as a natural product, limiting the control of safety and restricting the knowledge of possible harmful risks associated with them.

Although there is no regulation specifically for Kombucha, the British Columbia-Centre Disease Control (BC-CDC) designed an assessment and safety plan concerning the analytical and chemical parameters, ingredients, and associated claims to foster a more controlled production of this product [71]. More recently, the Canadian government has shown to be concerned about these novel products and has created different guidelines that help to assess the production process’ safety. These guidelines include the characterization of the Kombucha recipe, potential hazards associated, alcohol content, and labelling requirements. Labelling must contain the alcohol value if its superior to 1.1% *v/v*, and it gives suggestions on how to control the quality and safety of the product, preventing potential hazards during the process, like hygiene and sanitation, pH monitorization and control, and packaging materials [71]. For instance, these guidelines address the safety of the strains being used, the veracity of the product, and human consumption validation of different and new products [69].

### 5.4. Oceania

#### New Zealand and Australia

New Zealand and Australia have a joint agency called the Food Standard Australia and New Zealand (FSANZ), responsible for accepting and regulating health claims for probiotic products [50,72]. In this part of the world, probiotics fall under the definition of functional foods. Unlike regular and conventional foods, they are supposed to offer physiological benefits beyond simple nutrition [52,72]. In these countries, probiotics are traditionally available in foods like yogurt. The product is covered by the Food Standard, which categorizes products according to their function, nutrition, and health claim set out by the Food Standard Code 1.7 [73].

The Australian and New Zealand food code regulates the current use of nutrients and health claims [50]. There are two types of health claims: general claims, which refer to a nutrient or substance present in a product and its effect on health, and high-level claims about a nutrient or substance and its effect on a specific disease [74]. The Nutrient Profiling Scoring Criterion (NPSC) is a system used to assess if a food or beverage is suitable for making a health claim based on its nutrient content and criteria [50]. However, a product cannot be considered if it is not consumed or traditionally viewed as food, and it does not mean that it is a therapeutic good. To be perceived as a therapeutic product, it needs to be accompanied by a therapeutic claim, and it needs to be viewed by the consumers as an option for therapeutic use, subsequently regulated by the Australia Regulatory Therapeutic Goods, in line with the Food Code [73]. However, this does not assure the consumer to rely on such products. These guidelines help to categorize probiotics as a food or therapeutic good, but it does not control the safety of the probiotic itself.

Thus, the literature shows that although some countries and regions already have presented probiotic regulations (Figure 2), most of them are confused and unorganized, with a few of them offering concrete guidelines about manufacturing and selling. In addition, most regions have very specified label regulation, allowing some or absolutely no health-related claims. Still, when it comes to controlling the product’s safety, there is nothing that producers can rely on. Therefore, an unharmonized probiotic market can represent a significant danger to humans facing the increasing consumption, mainly because there is no precise control of the different analytical parameters used in the product production, which is a critical aspect to ensure most of the expected benefits.

Specifically, regarding Kombucha, a promising probiotic beverage that has invaded consumer markets and become more and more popular due to its good health benefits, it is urgent that governments focus on defining regulations to guarantee its quality and safety for both artisanal and industrial production [75].

## 6. Kombucha: S.W.O.T. Analysis

**Figure 3:**
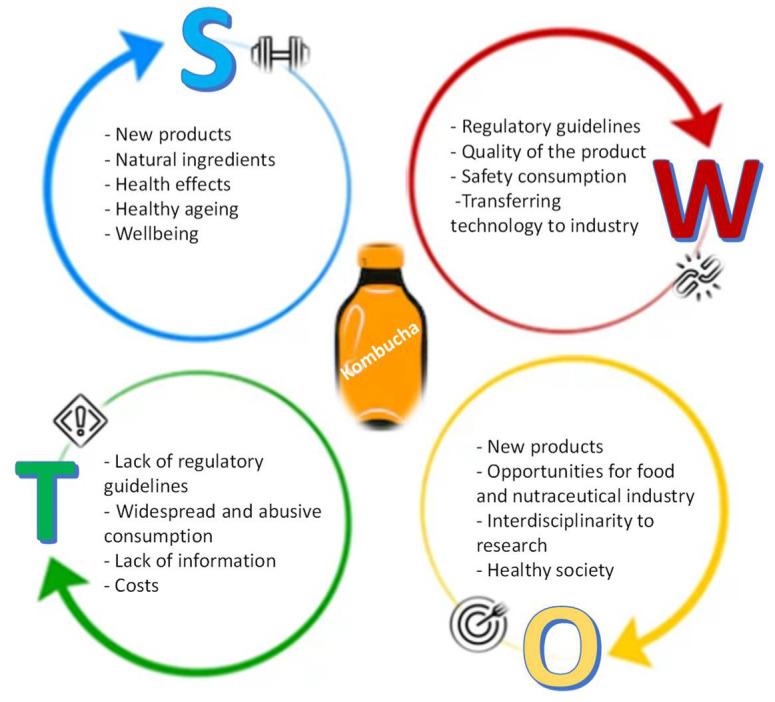
S.W.O.T. analysis for Kombucha market.

The search for healthier products that impact on health has been a challenge for researchers because most studies are performed in short-term frames, and longer studies are needed. It is difficult to identify short-term data because the benefit/impact is smooth, and on the other hand, the data affect the synergy between different systems and not each system, because there are many individual variables that interfere with these benefits. Another challenge of research in this area has to do with the lack of human studies. Most studies are conducted on animals, which makes difficult to correlate the positive and negative outcomes to humans. The ones that approach human subjects do not consider several variables, because they mostly occur in controlled environments without reflecting on all systems, whether individually or combined, which affects the human response, perception, and even the physiological system itself. Facing the significant impact of Kombucha on the health-related market and its growing popularity, it is helpful to reflect on this product and, hence, to consider a S.W.O.T. analysis (Strengths–Weaknesses–Opportunities–Threats). This analysis could allow the development of insights and interpretations about the product production for subsequent implementation in industry and society (Figure 3).

### 6.1. Strenghts

Modern society is becoming increasingly aware of health promotion policies, especially those related to healthy ageing [1,36]. Consequently, government and health authorities are increasingly concerned with developing and implementing sustainable policies (SDGs). On the other hand, citizens are trying to adopt new diet courses and new food to prevent some health problems, improve physical and mental performance, and demand more from the regulators. Kombucha has attracted attention exactly due to its wide range of applications as natural or functional food, primarily driven by a current consumer’s tendency toward healthier habits [19,20,21,25].

### 6.2. Weaknesses

The regulatory hurdles are the major weaknesses of the kombucha market, because consumers are calling into question the quality of the product, safety, and its acceptability. The technology or the cost of transferring the new technology to the food industry may be another obstacle. Furthermore, on one side, the lack of information about available products is a critical issue. However, on the other side, it is still also important to invest in the health literacy of probiotic products. We strongly believe that it is of crucial importance to allow representatives from academia, industry, consumers, and government to discuss, assess, and share experiences and best practices around improving Kombucha products’ quality and safety. These four actors can represent a new motor of innovation for Kombucha products. The ever-increasing competitive landscape, the speed of technological innovations, the shorter product life cycles, and the increase in demands from consumers require structured and clear regulation to be on top of new developments in this field. Accordingly, regulatory agencies, policymakers, industries, and researchers involved in the Kombucha products must collaborate to establish a quality standard, because any variation can directly impact product yields; costs; availability; and, most importantly, consumer safety.

### 6.3. Opportunities

There are great potentials and opportunities for the food and nutraceutical industry. Clearly, the transference of knowledge from research centers to businesses will be an added value and a path to follow [75].

Here, researchers, as the source of new knowledge and technology, could have an essential role. In transforming their roles and opening to other partnerships, they have deliberately invested time and efforts in developing a connective tissue with industries in order to achieve the most significant possible advances. These initiatives can foster more research in biotechnology and strengthen the collaboration between researchers, industries’ consumers, and health professionals. This interdisciplinarity can improve this product’s processing and create novel functional foods with high consumer acceptability.

Another big opportunity is the possibility of creating more channels through the promotion of safe products to foster health promotion and avoid health costs. The regulatory guidelines implementation will allow the monitoring of the product production as well as the guarantee of its safety, quality, and effectiveness.

### 6.4. Threats

As the global population is increasingly exposed to various health threats, the demands for health-enhancing foods are growing at a significantly faster rate, which can explain the increase in Kombucha’s consumption around the world. In the face of such a reality, this paper has covered the most significant threat in this field: the lack of regulations.

Although this is a knowledge-based society, there is still the fear related to the widespread consumption of these products, namely by people who cannot consume them, such as immuno-depressed patients or those with pathologies that may be compromised. The product price and the lack of information may also limit consumer acceptance.

## 7. Conclusions

In summary, new food products with an impact on human health and wellbeing are needed. Improving citizens’ health and healthy aging is an SGD aim. One way to achieve this aim is raising people’s awareness of health prevention and promotion through a more balanced diet, and the consumption of natural products that promotes health is fundamental.

More recently, the consumption of probiotics to protect and shape our intestinal microbiota has been increasingly studied. Consequently, innovative products have been developed, such as Kombucha. However, there is still a long way to go, especially when it comes to safe implementation on the market. Furthermore, the lack of regulation may limit its consumption, since it may compromise the quality and safety of the product.

This manuscript intends to draw attention to the urgency for regulatory guidelines for Kombucha processing and marketing to improve the quality and safety of the product as well as the need for action by governments, policymakers, and industries.

## Data Availability

The data presented in this study are available on request from the corresponding author.
